# Synergistic Effect of the Mucosa-Friendly Agents Berberine and Tea Tree Oil on Mucosal Protection Against *Neisseria gonorrhoeae* in an In Vitro T84 Cell Mucosa Model

**DOI:** 10.3390/antibiotics15040392

**Published:** 2026-04-12

**Authors:** Mon-Der Cho, Shang-Yu Chou, Jung-Sheng Chen, Yu-Ming Hsu, Chi-Ying Li, Yi-Hong Tsai, Fang-Rong Chang

**Affiliations:** 1 Graduate Institute of Natural Products, Kaohsiung Medical University, Kaohsiung 807378, Taiwan; u105831003@kmu.edu.tw; 2 Department of Urology, Kaohsiung Municipal Min-Sheng Hospital, Kaohsiung 802511, Taiwan; 3 Department of Radiation Oncology, Kaohsiung Chang Gung Memorial Hospital, Kaohsiung 833401, Taiwan; a9682@adm.cgmh.org.tw; 4 Department of Medical Research, E-Da Hospital, I-Shou University, Kaohsiung 824005, Taiwan; ed113187@edah.org.tw; 5 Department of Medical Science and Biotechnology, I-Shou University, Kaohsiung 824005, Taiwan; 6 Department of Pharmacy and Master Program, College of Pharmacy and Health Care, Tajen University, Pingtung 907101, Taiwan; maiz538272@gmail.com; 7 Research Center for Precision Environmental Medicine, Kaohsiung Medical University, Kaohsiung 807378, Taiwan; 8 School of Pharmacy, Tzu Chi University, Hualien 970374, Taiwan; cyli@gms.tcu.edu.tw; 9 Department of Marine Biotechnology and Resources, National Sun Yat-sen University, Kaohsiung 804201, Taiwan; 10 Drug Development and Value Creation Research Center, Kaohsiung Medical University, Kaohsiung 807378, Taiwan; 11 Department of Medical Research, Kaohsiung Medical University Hospital, Kaohsiung Medical University, Kaohsiung 807378, Taiwan

**Keywords:** berberine (BB), drug-resistant infections, *Neisseria gonorrhoeae*, synergistic antimicrobial effect, tea tree oil (TTO)

## Abstract

**Introduction:** *Neisseria gonorrhoeae*, a bacterium responsible for gonorrhea, can spread through oral sex, causing pharyngeal gonorrhea, which is also a leading cause of urethritis in outpatient clinics. This study investigated whether tea tree oil (TTO) alone or in combination with other natural products could serve as an effective alternative to chlorhexidine in preventing the spread of oral gonorrhea. **Methods:** An in vitro model was developed using T84 epithelial cells as a mucosal layer and *Neisseria gonorrhoeae* strain MS11. The study assessed the minimal inhibitory concentration (MIC), bacterial adherence, invasion, and transmigration across the mucosal barrier. Berberine (BB), a major and bioactive alkaloid derived from *Coptis chinensis*, was tested with and without TTO in MIC assays and epithelial cell viability tests. Ceftriaxone was used as a positive control. **Results:** The MIC values for TTO, BB, and ceftriaxone against the MS11 strain were determined to be 0.2%, 5 μg/mL, and 0.0125 μg/mL, respectively. Notably, the combination of TTO with BB demonstrated a synergistic effect, reducing the MIC to 0.000625% TTO + 1.25 μg/mL BB. This combination provided the strongest protective effect. No cytotoxicity was observed in the epithelial cell viability tests for 0.2% diluted TTO, 5 μg/mL BB, or the combination of 0.000625% TTO + 1.25 μg/mL BB. **Conclusions:** BB, when combined with TTO, exhibited a synergistic antimicrobial effect against *Neisseria gonorrhoeae* strain MS11 in the T84 mucosal model. These findings highlight the potential of this combination as a natural alternative to chlorhexidine gluconate for managing oral gonorrhea.

## 1. Introduction

Gonorrhoea remains a major global sexually transmitted infection and an important public health challenge [1,2,3,4]. According to the World Health Organization (WHO), an estimated 82.4 million new gonorrhoea cases occurred worldwide in 2020 [2]. Untreated infection can lead to serious complications, including pelvic inflammatory disease, infertility, and increased risk of onward transmission [2]. Of particular concern, pharyngeal gonorrhoea is more difficult to eradicate than urogenital or anorectal infection and may contribute to ongoing community transmission and antimicrobial resistance [3]. Current CDC (Centers for Disease Control and Prevention, USA) guidance recommends ceftriaxone as the first-line treatment, and no reliable alternative treatment is available for pharyngeal gonorrhoea [3]. In parallel, recent surveillance and meta-analytic data indicate that antimicrobial resistance in *Neisseria gonorrhoeae* remains a major global concern, underscoring the need for adjunctive or topical preventive strategies [2,3,4]. The causative agent *Neisseria gonorrhoeae* is a Gram-negative diplococcus with a kidney-shaped appearance, growing optimally at 35–37 °C in blood or chocolate agar under 3–10% CO_2_ [5].

**Figure 1:**
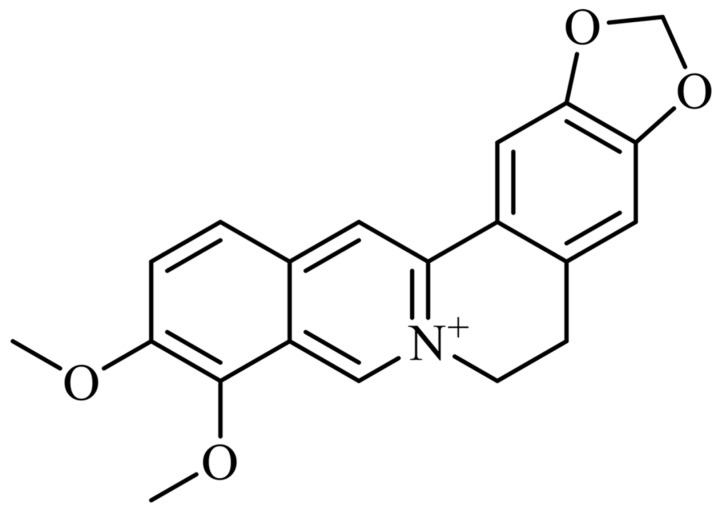
The chemical structure of berberine (BB): a benzylisoquinoline alkaloid found in *Berberis* species. Its planar aromatic structure contributes to its antimicrobial and biological activities.

Berberine (BB), as shown in Figure 1, is a plant-derived isoquinoline alkaloid with broad-spectrum antimicrobial activity [6,7]. Recent evidence suggests that its antibacterial actions may involve interference with efflux-associated resistance, disruption of bacterial division proteins such as FtsZ, impairment of membrane and cell-wall integrity, and inhibition of DNA/RNA-related processes [6,7]. In *Neisseria gonorrhoeae*, previous susceptibility studies have demonstrated measurable in vitro activity of berberine against both reference and clinical isolates [8]. However, the clinical application of berberine as a standalone antimicrobial remains limited by poor bioavailability and insufficient in vivo validation [6,7]. These limitations make berberine an attractive candidate for combination strategies, particularly in topical or mucosal formulations where synergistic enhancement may reduce the concentration required for activity [6,7,8,9].

Tea tree oil (TTO), distilled from *Melaleuca alternifolia*, is a complex essential oil widely used in topical antimicrobial preparations. Its major biologically relevant constituents include terpinen-4-ol, γ-terpinene, and 1,8-cineole, and current evidence supports membrane-active antimicrobial effects as a central mechanism underlying its antibacterial activity [10,11,12,13]. In addition to its broad in vitro antimicrobial profile, recent human evidence indicates that the efficacy and safety of tea tree oil are formulation-dependent, and mucosal or topical tolerability should be interpreted cautiously, particularly at higher concentrations or with oxidized preparations [13]. These properties nevertheless make TTO a rational candidate for combination strategies aimed at enhancing local antimicrobial activity [10,11,13].

This study aimed to evaluate whether TTO, alone or in combination with berberine, exerts synergistic antigonococcal activity against *N. gonorrhoeae* in an in vitro T84 mucosal model. We focused on the laboratory strains MS11 WT and MS11 Δopa, the latter lacking Opa proteins involved in bacterial adherence and immune interaction [14,15]. By examining MIC reduction, bacterial adhesion, killing activity, and host–cell cytotoxicity, we sought to assess whether the TTO–BB combination could serve as a safer topical adjunct for reducing mucosal colonization and transmission risk.

## 2. Results

### 2.1. Chemical Composition and Fingerprint Analysis of TTO

**Figure 2:**
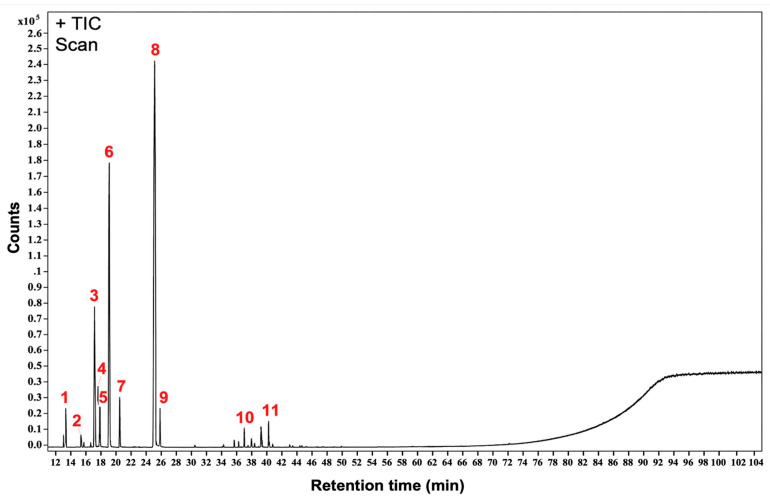
Gas chromatography–mass spectrometry fingerprint of TTO. The chromatogram highlights the major chemical constituents of TTO, including α-pinene (1), sabinene (2), α-terpinene (3), *p*-cymene (4), eucalyptol (5), γ-terpinene (6), terpinolene (7), terpinen-4-ol (8), α-terpineol (9), aromadendrene (10), and β-cadinene (11). Among these, terpinen-4-ol (8), γ-terpinene (6), and α-terpinene (3) are the most abundant, representing the primary active components. This fingerprint serves as a reference for quality control and chemical manufacturing processes of TTO.

The chemical fingerprint of TTO for chemical manufacturing control is illustrated in Figure 2, providing a comprehensive profile of its constituents. The chemical composition of TTO was analyzed using gas chromatography–mass spectrometry (GC-MS), revealing the presence of the following compounds: α-pinene (1), sabinene (2), α-terpinene (3), *p*-cymene (4), eucalyptol (5), γ-terpinene (6), terpinolene (7), terpinen-4-ol (8), α-terpineol (9), aromadendrene (10), and β-cadinene (11). Among these constituents, terpinen-4-ol (8), γ-terpinene (6), and α-terpinene (3) were identified as the most abundant, collectively representing the key active components of TTO. These dominant compounds contribute significantly to the biological and therapeutic properties of TTO, particularly its antimicrobial and anti-inflammatory effects. The detailed chemical profile outlined in Figure 2 and Table 1 serves as a robust framework for quality assurance in the standardization of TTO during its manufacturing process. By establishing a reproducible fingerprint, manufacturers can ensure batch-to-batch consistency, maintaining the efficacy and safety of TTO in its various applications. The relative abundance of the identified constituents confirms that the TTO evaluated in this experiment conforms to the specifications of the ISO 4730:2017 standard [12,16].

### 2.2. Synergistic Antimicrobial Effect of BB and TTO Against Neisseria gonorrhoeae

**Figure 3:**
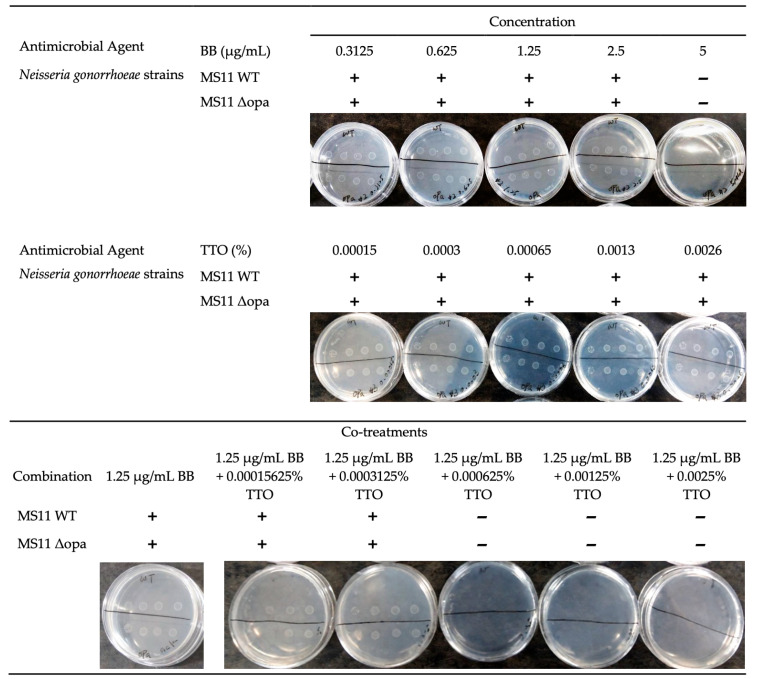
Synergistic antimicrobial activity of BB, TTO, and their combination against *Neisseria gonorrhoeae* strains MS11 WT and MS11 Δopa. The top panel shows the inhibitory effect of serially diluted BB alone (µg/mL), while the middle panel displays the activity of serially diluted TTO alone (*v*/*v* %). The bottom panel illustrates the combination treatment, demonstrating the effect of varying TTO concentrations combined with a fixed concentration of BB (1.25 µg/mL). The presence (+) or absence (−) of visible bacterial growth on the agar plates determines the minimum inhibitory concentration (MIC). The combination treatment demonstrates a significantly enhanced inhibitory effect, completely eradicating bacterial growth at concentrations significantly lower than their individual MICs (achieving synergy at 1.25 µg/mL BB + 0.000625% TTO). This visual evidence highlights the potent synergistic bactericidal potential of the BB-TTO mixture. The data represent one of three independent experiments with identical results. For quantitative synergistic analysis and statistical significance (*p* < 0.05), please refer to Table 2.

In Table 2, BB and TTO exhibited MIC values of 5 μg/mL and 0.20%, respectively, against both GC strains, MS11 WT and MS11 Δopa. To provide direct visual evidence of this inhibitory effect, the growth inhibition profiles on agar plates are presented in Figure 3. Notably, when BB and TTO were combined, the treatment demonstrated a potent synergistic effect, completely eradicating bacterial growth at concentrations significantly lower than their individual MICs (achieving synergy at 1.25 μg/mL BB + 0.000625% TTO). The Fractional Inhibitory Concentration Index (FICI) was calculated to be 0.250, which is well below the standard threshold (FICI ≤ 0.5), mathematically confirming the strong synergistic interaction of the BB-TTO mixture. This substantial reduction in effective concentrations highlights the notable potential of the mixture, especially when compared to the standard antibiotic ceftriaxone, which exhibited an MIC of 0.0125 μg/mL for both strains. The data indicate that the combination of BB and TTO enhances antimicrobial efficacy, potentially due to complementary mechanisms of action. While BB likely disrupts bacterial metabolism or DNA processes, TTO, known for its membrane-disrupting properties, may facilitate BB’s penetration into bacterial cells, amplifying its inhibitory effects. The MIC values for the MS11 Δopa mutant, which lacks opa proteins involved in bacterial adherence and immune evasion, were comparable to those of the wild-type strain (MS11 WT). This suggests that the synergistic effect of BB and TTO is not significantly influenced by the opa proteins. These findings demonstrate the potential application of the BB-TTO combination as a natural antimicrobial strategy for treating drug-resistant gonorrhea.

### 2.3. Safety and Enhanced Antimicrobial Activity of the TTO-BB Combination in Disrupting Bacterial Colonization

**Figure 4:**
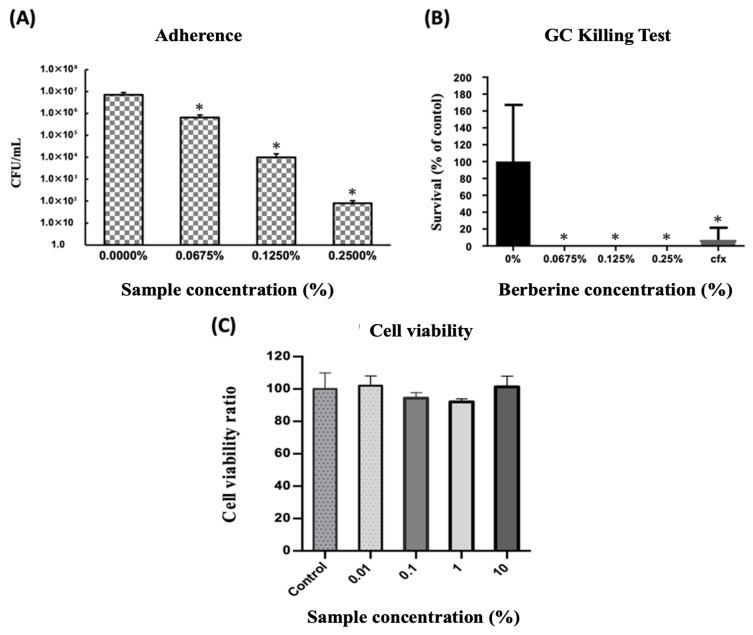
Safety and enhanced antimicrobial activity of the TTO-BB combination in disrupting bacterial colonization. (**A**) The TTO-BB mixture significantly reduced GC adhesion to T84 epithelial cells in a concentration-dependent manner, effectively disrupting bacterial colonization. (**B**) The combination eradicated GC survival at all tested concentrations, showing marked bactericidal activity under the tested assay conditions, particularly at lower concentrations. (**C**) No cytotoxic effects were observed on T84 cells, even at 10% concentration, underscoring the safety of the TTO-BB mixture as a therapeutic agent. Data are expressed as the mean ± standard deviation (SD) from three independent replicates. Statistical significance was determined by one-way ANOVA followed by Tukey’s post hoc test. Asterisks * indicate significant differences (*p* < 0.05) compared with the control group (0% or Control).

Figure 4 demonstrates the potent antimicrobial activity and safety profile of the TTO and BB combination against GC, showcasing its potential as a natural therapeutic agent. In Figure 4A, the TTO-BB mixture significantly inhibited GC adhesion to T84 epithelial cells in a concentration-dependent manner, effectively disrupting a crucial step in bacterial colonization and infection establishment. This anti-adhesion property highlights its ability to target early infection stages and prevent bacterial persistence on host tissues. Meanwhile, Figure 4B showed that the TTO-BB combination markedly reduced GC survival across the tested conditions, demonstrating strong bactericidal activity in this assay. Remarkably, its bactericidal efficacy surpassed ceftriaxone, a standard antibiotic, particularly at lower concentrations, underscoring the synergistic interaction between TTO and BB. The enhanced activity likely results from TTO’s membrane-disrupting properties, facilitating BB’s intracellular action, amplifying its antimicrobial effects while reducing the effective concentrations required. Furthermore, Figure 4C confirms the safety of the combination, showing no cytotoxicity on T84 epithelial cells, even at the highest concentration tested (10%). This ensures that the TTO-BB mixture can be used safely without damaging host tissues. Collectively, these findings highlight the dual benefits of the TTO-BB combination, combining strong antimicrobial efficacy with excellent safety. The superior performance compared to ceftriaxone and the absence of cytotoxic effects make it a promising natural alternative or adjunctive therapy for addressing the growing issue of drug-resistant gonorrhea.

## 3. Discussion

Using the in vitro T84 mucosal model, this study investigated various aspects of gonorrheal infection, including MIC profiles, bacterial adhesion, and killing assays. The combination of TTO and BB demonstrated significantly enhanced efficacy, reducing the concentration required to inhibit *N. gonorrhoeae* compared with either agent alone. Specifically, the effective inhibitory concentration of BB in the mixture decreased from 5 μg/mL to 1.25 μg/mL, and that of TTO was reduced from 0.20% to 0.000625%, mathematically confirming a strong synergistic effect (FICI = 0.250). Beyond MIC reduction, the combination also impaired bacterial adhesion to epithelial cells and markedly reduced bacterial survival in the killing assay. Importantly, no overt cytotoxicity was observed under the tested conditions (Figure 4), supporting the feasibility of this combination for further evaluation in mucosal applications. These findings suggest that the benefit of the combination extends beyond growth inhibition alone and may effectively disrupt early colonization-related events.

The GC–MS analysis showed that the tested tea tree oil was rich in terpinen-4-ol, γ-terpinene, and α-terpinene, which is broadly consistent with currently recognized biologically active TTO preparations [10,11,12]. In addition to supporting mechanistic plausibility, the detailed chemical fingerprint (Figure 2, Table 1) provides a baseline reference profile for future quality control. Routine monitoring of these major active components can help evaluate batch-to-batch consistency during manufacturing, thereby supporting the safety and efficacy of TTO in therapeutic applications.

The present findings are consistent with prior reports showing that berberine has measurable in vitro activity against *N. gonorrhoeae* and other bacterial pathogens [6,7,8]. Recent reviews further suggest that berberine acts through multiple antibacterial mechanisms, including interference with efflux-associated resistance, disruption of bacterial division, and inhibition of DNA/RNA-related processes [6,7]. At the same time, berberine has recognized translational limitations, especially low bioavailability, which reduces its attractiveness as a standalone systemic antimicrobial [7]. In this context, the combination approach used here is highly rational, as synergistic pairing improves antibacterial performance while significantly lowering the concentration of berberine required for activity.

TTO is also supported by substantial in vitro evidence as a broad-spectrum antimicrobial, with membrane disruption and altered cell-envelope integrity considered key contributors to its activity [10,11]. The synergistic interaction observed between TTO and berberine in this study is biologically plausible. A reasonable interpretation is that the membrane-active properties of TTO (particularly driven by terpinen-4-ol) may facilitate bacterial exposure or intracellular access to berberine, while berberine contributes additional intracellular or resistance-modifying effects [9,17]. Importantly, the combination remained active against the MS11 Δopa mutant, suggesting that the observed antimicrobial interaction was not dependent on Opa expression status alone [14,15]. However, these mechanisms were not directly tested in the present work and should therefore be interpreted as hypotheses rather than demonstrated mechanisms.

Taken together, our findings support the TTO–BB combination as a promising candidate for further development as a topical or mucosal adjunct against gonococcal colonization. Nevertheless, several limitations should be acknowledged. First, this study was conducted in an in vitro T84 model and included only laboratory strains (MS11 WT and Δopa), and therefore, may not fully reflect the behavior of contemporary clinical or highly resistant isolates at pharyngeal mucosal sites [1,3,4]. Second, the lack of experimental data regarding the efficacy of fully developed commercial formulations (e.g., mouthwashes or topical gels) constitutes a limitation of the current study. Further in vivo studies, clinical trials, and the evaluation of standardized commercial formulations are necessary to validate these findings and establish the therapeutic applicability of the TTO-BB combination in managing drug-resistant sexually transmitted infections.

## 4. Materials and Methods

### 4.1. GC-MS Analyses

The qualitative analysis of TTO was performed using an Agilent 7820/5977B GC-MS system (Agilent Technologies, Santa Clara, CA, USA). The commercial Australian TTO sample (manufactured by J C Buck Ltd., Brentwood, UK), provided by Healthcare Biotechnology Inc. (Kaohsiung, Taiwan), was diluted to 10% (*v*/*v*) in *n*-hexane. A 1 μL aliquot of the diluted sample was injected using an Agilent 7693A autoinjector. The injector was maintained at 280 °C and operated in split mode with a split ratio of 40:1. Separation was achieved on an HP-5MS capillary column (30 m × 0.25 mm × 0.25 µm, Agilent Technologies, Santa Clara, CA, USA). High-purity helium (99.999%) was used as the carrier gas at a constant flow rate of 1.0 mL/min. The oven temperature program was initiated at 60 °C, increased to 250 °C at a heating rate of 2 °C/min, and held at 250 °C for 10 min, resulting in a total run time of 105 min.

For the mass spectrometer, electron ionization (EI) was operated at 70 eV, with an ion source temperature of 230 °C and a mass scan range of *m*/*z* 40–500. Mass spectra were analyzed using Agilent MassHunter Workstation Software (version B.07.00). Compound identification was achieved by comparing the mass spectra with reference data from the Wiley/NBS Registry (version 5.0) and the NIST MS Search (version 2.0) databases. Kovats indices (KIs) and retention indices (RIs) were calculated relative to a homologous series of n-alkanes (*C*_8_–*C*_20_, Sigma-Aldrich, St. Louis, MO, USA) analyzed under identical operating conditions. Additionally, spectral data and RI values were cross-referenced with the literature, specifically Adams’ Identification of Essential Oil Components [16].

### 4.2. Antimicrobial Susceptibility Testing Using BB

**Figure 5:**
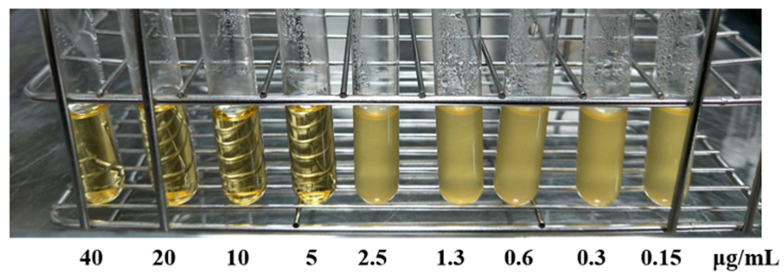
MIC assessment of Carbapenem-resistant *Klebsiella pneumoniae* (CRKP) using serial dilutions of BB in Mueller–Hinton Broth.

Commercial berberine (BB) extract powder (purity ≥ 98%) was purchased from Vital Herbs, Delhi, India. To evaluate the antimicrobial activity of BB, serial dilutions were prepared in Mueller–Hinton Broth (Sigma-Aldrich, St. Louis, MO, USA). To evaluate the antimicrobial activity of BB, serial dilutions were prepared in Mueller–Hinton Broth. BB was diluted to achieve final concentrations of 4, 2, 1, 0.5, 0.25, 0.13, 0.065, 0.0312, and 0.015 μg/mL, resulting in a total of nine concentration levels. Each concentration was dispensed into sterile glass test tubes with a total volume of 3 mL per tube (shown in Figure 5). Bacterial cultures of Group B *Streptococcus* (GBS), Carbapenem-Resistant *Acinetobacter baumannii* (CRAB), Carbapenem-Resistant *Klebsiella pneumoniae* (CRKP), and Carbapenem-Resistant *Escherichia coli* (CRE) were prepared at an inoculum density of 5 × 10^5^ CFU/mL. These bacterial suspensions were added to the corresponding test tubes containing BB dilutions. Following inoculation, the test tubes were incubated at 37 °C for 16–18 h. Post-incubation, visual and spectrophotometric assessments were conducted to identify the lowest concentration of BB that effectively inhibited bacterial growth, thereby determining the MIC for each strain (presented in Table 3).

### 4.3. Combination Antimicrobial Susceptibility Testing and FICI Calculation

To evaluate the potential synergistic antimicrobial effect of BB and TTO in combination, the fractional inhibitory concentration index (FICI) was determined. The MIC values of BB and TTO, both alone and in combination, were obtained through standard broth microdilution or agar dilution methods. The interaction between the two antimicrobial agents was quantified using the following FICI formula:

FICI = (MIC of BB in combination/MIC of BB alone) + (MIC of TTO in combination/TTO alone)

The interaction profiles were interpreted based on strictly established criteria: FICI ≤ 0.5 indicates a synergistic effect; 0.5 < FICI ≤ 1 indicates an additive effect; 1 < FICI ≤ 4 indicates indifference (no interaction); and FICI > 4 indicates antagonism.

### 4.4. Adherence to T84 Cells

Different concentrations of BB were prepared and added to the MS11 Δopa strain (seeding concentration 5 × 10^7^). It was placed in a T84 Transwell with a TEER value greater than 1000 mΩ, and then placed in an incubator and left to stand for 3 h (37 °C; 5% CO_2_). After 3 h, it was taken out and soaked in saponin, and shaken for 15 min. After that, it was taken out for serial dilution and spread on the plate. After coating the plate, it was placed in an incubator (37 °C, 5% CO_2_) and left to stand. After 16 h, it was taken out to calculate the colony count and reduce the original bacterial count.

### 4.5. Killing Assay

A killing assay was conducted to evaluate the antimicrobial efficacy of various concentrations of BB combined with TTO and ceftriaxone (MIC: 0.0125 μg/mL) against GC. The bacterial culture was prepared at an initial inoculum of 10^7^ CFU/mL and added to a 24-well plate containing the test compounds. The plates were incubated at 37 °C in a 5% CO_2_ environment for 3 h. At predetermined time points, aliquots were collected, serially diluted, and spread onto agar plates. The plates were incubated for an additional 16 h under the same conditions. Colony counts were then recorded, and the total bacterial population was calculated based on the dilution factor. This allowed for the quantification of bacterial survival and determination of the bactericidal activity of the test compounds.

### 4.6. Survival Test

To evaluate the cytotoxicity of BB, T84 cells were seeded onto a 96-well plate at a density of 10,000 cells per well. The plate was incubated at 37 °C in a humidified atmosphere with 5% CO_2_ for 24 h to allow cell adhesion. After incubation, the culture medium was removed and replaced with fresh medium containing various concentrations of BB. The plate was returned to the incubator for an additional 24 h. Following the exposure period, 10 μL of Cell Counting Kit-8 (CCK-8) reagent was added to each well, and the plate was incubated for 1 h under the same conditions. Absorbance was measured at 450 nm using a microplate reader. The survival rate of cells was calculated as the ratio of the absorbance of the experimental group to that of the control group. The cell culture medium used was DMEM/F12 supplemented with 7% fetal bovine serum (FBS) and 1% penicillin–streptomycin (P/S), and all experiments were conducted under standard culture conditions (37 °C, 5% CO_2_).

### 4.7. Statistical Analysis

Experimental data are expressed as mean ± standard deviation (SD) from at least three independent replicates. Statistical significance was determined using one-way ANOVA followed by Tukey’s post hoc test for multiple-group comparisons, or an unpaired Student’s *t*-test for comparisons between two groups. A *p*-value < 0.05 was considered statistically significant.

## 5. Conclusions

This study confirms the synergistic antimicrobial effect of TTO and BB against *Neisseria gonorrhoeae*, significantly reducing the minimum inhibitory concentrations of both agents. The TTO-BB combination demonstrated enhanced bactericidal activity, disrupted bacterial adhesion, and maintained a high safety profile, with no cytotoxic effects observed on host cells. These findings highlight the promising application of this combination in treating gonorrheal infections, particularly in the context of increasing antibiotic resistance. Furthermore, the reproducible chemical fingerprint of TTO and the established antimicrobial mechanisms of BB underscore their potential as natural therapeutic alternatives or adjuncts to conventional treatments. However, the lack of experimental data regarding the efficacy of fully developed commercial formulations (e.g., mouthwashes or topical gels) constitutes a limitation of the current study. Further in vivo studies, clinical trials, and the evaluation of standardized commercial formulations are necessary to validate these results and establish the therapeutic applicability of the TTO-BB combination in managing drug-resistant sexually transmitted infections. This research emphasizes the value of integrating traditional natural products to develop innovative and effective strategies against global health challenges.

## Figures and Tables

**Table 1:** Chemical constituents and major quality-control markers of TTO identified by GC-MS.

No.	Compound Name	R.t. (min) ^a^	KI (RI) ^b^	RI Values from the Literature ^c^	% RA (Relative Area) ^d^	Identification ^c^ (MS in Matching Scores)
1	α-pinene	13.191	935	939	2.53%	KI, MS (98.0)
2	sabinene	15.162	974	975	1.01%	KI, MS (92.9)
3	α-terpinene	16.864	1014	1017	10.52%	KI, MS (98.3)
4	*p*-cymene	17.202	1022	1024	4.13%	KI, MS (96.7)
5	eucalyptol	17.638	1031	1031	3.38%	KI, MS (93.2)
6	γ-terpinene	18.794	1058	1059	21.16%	KI, MS (98.5)
7	terpinolene	20.111	1086	1088	4.67%	KI, MS (96.6)
8	terpinen-4-ol	24.771	1175	1177	41.88%	KI, MS (97.5)
9	α-terpineol	25.366	1187	1188	3.72%	KI, MS (97.9)
10	aromadendrene	36.529	1438	1441	3.12%	KI, MS (93.5)
11	β-cadinene	39.660	1507	1513	3.88%	KI, MS (94.8)

^a^ Retention time (min). ^b^ KI (Kovats indices): Retention indices (RI) relative to *n*-alkanes (*C*_8_–*C*_20_) on HP-5MS column. ^c^ Identification based on KI on a HP-5MS column in reference [16], along with comparison with reference data from the Wiley/NBS Registry of Mass Spectral Data (version 5.0) and the National Institute of Standards and Technology (NIST) MS Search (version 2.0). ^d^ Relative area ratio expressed as internal normalization of the 11 identified peaks.

**Table 2:** MIC values of BB, TTO, and their combination (TTO + BB) against *Neisseria gonorrhoeae* strains MS11 WT and MS11 Δopa, showing a synergistic effect.

Bacterial Strain	BB MIC (µg/mL)	TTO MIC (%)	FICI, Fractional Inhibitory Concentration Index	Ceftriaxone MIC (µg/mL)
*Neisseria gonorrhoeae*			(Synergy at 1.25 µg/mL BB +0.000625% TTO)	
MS11 WT	5.000 ± 0.117	0.200 ± 0.009	0.250 ± 0.018 *	0.0125 ± 0.0020
MS11 Δopa	5.000 ± 0.328	0.200 ± 0.016	0.250 ± 0.031 *	0.0125 ± 0.0060

Data are presented as the mean ± standard deviation (SD) of three independent experiments (n = 3). * *p* < 0.05 indicates a statistically significant difference compared with the individual agents alone, determined by one-way ANOVA followed by Tukey’s post hoc test. FICI ≤ 0.5 indicates a synergistic effect.

**Table 3:** MIC values of BB against bacterial strains, determined after 16–18 h incubation at 37 °C in Mueller–Hinton Broth.

Multidrug-Resistant Bacterial Strain	MIC (μg/mL)
Group B. *Streptococcus* (GBS)	0.062 ± 0.037
Carbapenem-Resistant *Acinetobacter baumannii* (CRAB)	0.500 ± 0.012
Carbapenem-Resistant *Klebsiella pneumoniae* (CRKP)	0.425 ± 0.065
*Escherichia coli* (CRE)	0.250 ± 0.083

Data are presented as the mean ± standard deviation (SD) of three independent experiments (n = 3).

## Data Availability

All data generated or analyzed during this study are included in this article. Further enquiries can be directed to the corresponding authors.

## Abbreviations

The following abbreviations are used in this manuscript: AIDS, acquired immunodeficiency syndrome; BB, berberine; CCK-8, Cell Counting Kit-8; CFU, colony-forming units; CRAB, carbapenem-resistant *Acinetobacter baumannii*; CRE, carbapenem-resistant Enterobacteriaceae; CRKP, carbapenem-resistant *Klebsiella pneumoniae*; DMEM, Dulbecco’s Modified Eagle Medium; FBS, fetal bovine serum; GC, gonococci (*Neisseria gonorrhoeae*); GC-MS, gas chromatography–mass spectrometry; GBS, Group B *Streptococcus*; MDR, multidrug resistance; MIC, minimum inhibitory concentration; MRSA, methicillin-resistant *Staphylococcus aureus*; P/S, penicillin–streptomycin; STDs, sexually transmitted diseases; TTO, tea tree oil; WHO, World Health Organization.

## References

[1] World Health Organization (2025). Multi-Drug Resistant Gonorrhoea.

[2] World Health Organization (2025). Gonorrhoea (Neisseria gonorrhoeae Infection).

[3] Centers for Disease Control and Prevention (2021). Gonococcal Infections Among Adolescents and Adults. STI Treatment Guidelines.

[4] Hooshiar M.H., Sholeh M., Beig M., Azizian K., Kouhsari E. (2024). Global trends of antimicrobial resistance rates in *Neisseria gonorrhoeae*: A systematic review and meta-analysis. Front. Pharmacol..

[5] Ng L.K., Martin I.E. (2005). The laboratory diagnosis of *Neisseria gonorrhoeae*. Can. J. Infect. Dis. Med. Microbiol..

[6] Duda-Madej A., Viscardi S., Bazan H., Sobieraj J. (2025). Exploring the Role of Berberine as a Molecular Disruptor in Antimicrobial Strategies. Pharmaceuticals.

[7] Yang X., Wang Y., Li L., Tang D., Yan Z., Li M., Jiang J., Bi D. (2025). Berberine and its nanoformulations and extracts: Potential strategies and future perspectives against multi-drug resistant bacterial infections. Front. Microbiol..

[8] Zheng X.L., Xu W.Q., Liu J.W., Zhu X.Y., Chen S.C., Han Y., Dai X.Q., Goodman I.G., Budjan C., Chen X.S. (2020). Evaluation of Drugs with Therapeutic Potential for Susceptibility of *Neisseria gonorrhoeae* Isolates from 8 Provinces in China from 2018. Infect. Drug Resist..

[9] Stermitz F.R., Lorenz P., Tawara J.N., Zenewicz L.A., Lewis K. (2000). Synergy in a medicinal plant: Antimicrobial action of berberine potentiated by 5′-methoxyhydnocarpin, a multidrug pump inhibitor. Proc. Natl. Acad. Sci. USA.

[10] Iacovelli F., Romeo A., Lattanzio P., Ammendola S., Battistoni A., La Frazia S., Vindigni G., Unida V., Biocca S., Gaziano R. (2023). Deciphering the Broad Antimicrobial Activity of *Melaleuca alternifolia* Tea Tree Oil by Combining Experimental and Computational Investigations. Int. J. Mol. Sci..

[11] Carson C.F., Hammer K.A., Riley T.V. (2006). *Melaleuca alternifolia* (Tea Tree) oil: A review of antimicrobial and other medicinal properties. Clin. Microbiol. Rev..

[12] (2017). Essential Oil of Melaleuca, Terpinen-4-ol Type (Tea Tree Oil).

[13] Kairey L., Agnew T., Bowles E.J., Barkla B.J., Wardle J., Lauche R. (2023). Efficacy and safety of *Melaleuca alternifolia* (tea tree) oil for human health—A systematic review of randomized controlled trials. Front. Pharmacol..

[14] LeVan A., Zimmerman L.I., Mahle A.C., Swanson K.V., DeShong P., Park J., Edwards V.L., Song W., Stein D.C. (2012). Construction and characterization of a derivative of *Neisseria gonorrhoeae* strain MS11 devoid of all opa genes. J. Bacteriol..

[15] Ball L.M., Criss A.K. (2013). Constitutively Opa-expressing and Opa-deficient *Neisseria gonorrhoeae* strains differentially stimulate and survive exposure to human neutrophils. J. Bacteriol..

[16] Adams R.P. (2001). Identification of Essential Oil Components by Gas Chromatography/Quadrupole Mass Spectroscopy.

[17] Raikwar G., Kumar D., Mohan S., Dahiya P. (2024). Synergistic potential of essential oils with antibiotics for antimicrobial resistance with emphasis on mechanism of action: A review. Biocatal. Agric. Biotechnol..

